# SIRT1-Mediated Mitochondrial Homeostasis in Cardiac Aging: Molecular Mechanisms and Therapeutic Implications

**DOI:** 10.14336/AD.2025.1128

**Published:** 2026-10-19

**Authors:** Sitong Chen, Hanying Xu, Xiaonan Li, Xiaolei Tang, Lan Yang, Jing Lu, Jun Li

**Affiliations:** ^1^College of Traditional Chinese Medicine, Changchun University of Chinese Medicine, Changchun, China.; ^2^Department of Encephalopathy, The Affiliated Hospital of Changchun University of Chinese Medicine, Changchun, China.; ^3^Geriatrics Department, The First Hospital of Jilin University, Changchun, China.; ^4^Research Center of Chinese Traditional Medicine, The Affiliated Hospital of Changchun University of Chinese Medicine, Changchun, China.; ^5^College of Integrated Traditional Chinese and Western Medicine, Changchun University of Chinese Medicine, Changchun, China.; ^6^Vascular Surgery, The Affiliated Hospital of Changchun University of Chinese Medicine, Changchun, China

**Keywords:** Sirtuin 1, mitochondrion, cardiac aging, cardiovascular disease, therapeutic strategies

## Abstract

Cardiovascular disease (CVD) remains the leading cause of global mortality and disability. As an inevitable risk factor, cardiac aging significantly exacerbates the incidence and progression of age-related cardiovascular pathologies, including coronary artery disease, cardiomyopathies, and heart failure in the elderly population. Mitochondria function as central organelles in cardiac energy metabolism. Dysregulation of functional homeostasis, characterized by impaired quality control mechanisms, such as diminished energy production efficiency and exacerbated oxidative stress, is a primary driver of the cardiac aging process. Accumulating evidence in recent years indicates that sirtuin 1 (SIRT1) plays a crucial role in regulating cardiac aging. A range of therapeutic agents, including natural compounds and synthetic molecules, ameliorate cardiac aging and related pathologies by activating SIRT1 to modulate mitochondrial function. This review systematically summarizes the emerging roles of SIRT1 in cardiac aging, with a focus on the molecular mechanisms through which SIRT1 governs mitochondrial homeostasis. We also highlight recent advances in SIRT1-targeted therapeutic strategies, thereby providing a theoretical basis and translational perspectives for preventing and treating cardiac aging-related diseases.

## 1. Introduction

Cardiovascular disease (CVD) is strongly linked to age-related physiological decline, with the aging of the global population serving as a principal driver behind the increasing burden of CVD [1]. Epidemiological studies have demonstrated that the prevalence of coronary artery disease is markedly greater in adults over 65 years of age compared to those aged 45-64, with the risk approximately doubling per decade of advancing age [2]. Cardiac aging represents a multifaceted patho-physiological process involving the progressive deterioration of cardiac structure and function. Hallmark features include a decline in cardiomyocyte number with compensatory hypertrophy, elevated myocardial interstitial fibrosis, and increased collagen cross-linking [3]. These alterations collectively contribute to heightened myocardial stiffness and reduced ventricular compliance. The resulting structural remodeling compromises active relaxation during diastole and diminishes cardiac reserve capacity, thereby substantially elevating the risk of cardiovascular events [4].

Mitochondrial dysfunction is a central mechanism driving the cardiac aging process [5]. As one of the most metabolically active organs in the body, the heart is highly dependent on ATP generated by mitochondria to sustain its contractile function and cellular homeostasis [6]. During cardiac aging, mitochondria exhibit multiple functional deficiencies, most notably a significant decline in ATP synthesis efficiency, which can precipitate a cellular energy crisis [7]. Furthermore, dysfunctional mitochondria overproduce reactive oxygen species (ROS), exacerbating oxidative damage within the myocardium [8]. Additionally, the accumulation of mitochondrial DNA (mtDNA) damage and impaired mitophagy leads to a substantial buildup of damaged mitochondria [9]. Collectively, these interrelated mitochondrial abnormalities act synergistically to promote the progression of cardiac aging [10].

Given the critical role of mitochondrial homeostasis in cardiac aging, targeting mitochondrial function has become a key therapeutic strategy for delaying this process. Sirtuin 1 (SIRT1) is a pivotal regulator of mitochondrial function. In the aging heart, the expression and activity of SIRT1 progressively decline [11]. Conversely, activating SIRT1 helps maintain mitochondrial homeostasis and improves cardiomyocyte function [12]. Therefore, the SIRT1-mitochondrial axis represents a promising target for intervention in cardiac aging. This review systematically elaborates on the core molecular mechanisms through which SIRT1 regulates mitochondrial homeostasis to ameliorate cardiac aging and discusses associated therapeutic strategies.

## 2. Fundamental biology of SIRT1

### 2.1 Structural basis and subcellular localization of SIRT1

SIRT1 is a class III histone deacetylase that is dependent on the cofactor nicotinamide adenine dinucleotide (NAD^+^) [13]. The human SIRT1 gene is located at chromosome 10q21.3 and encodes a 747-amino acid multidomain protein [14]. The protein comprises three functional domains: an N-terminal regulatory domain (residues 1-243), a central catalytic core domain (residues 244-512), and a C-terminal regulatory domain (residues 513-747) [15]. The catalytic core domain consists of two structurally distinct subdomains: a large Rossmann fold subdomain that recognizes and binds the NAD^+^ cofactor through conserved residues, and a zinc-binding subdomain that maintains conformational stability via tetrahedral coordination of Zn²^+^ ions by cysteine residues [16]. These subdomains cooperatively form the substrate-binding catalytic pocket, which serves as the functional core for enzymatic activity [17]. Additionally, the N- and C-terminal regulatory domains undergo intramolecular folding to engage the catalytic core through allosteric interactions, substantially enhancing the catalytic efficiency of SIRT1. Incorporated nuclear localization signals (NLSs) and nuclear export signals (NESs) within these terminal domains further regulate subcellular trafficking dynamics [18] (Fig. 1).

**Figure 1.**
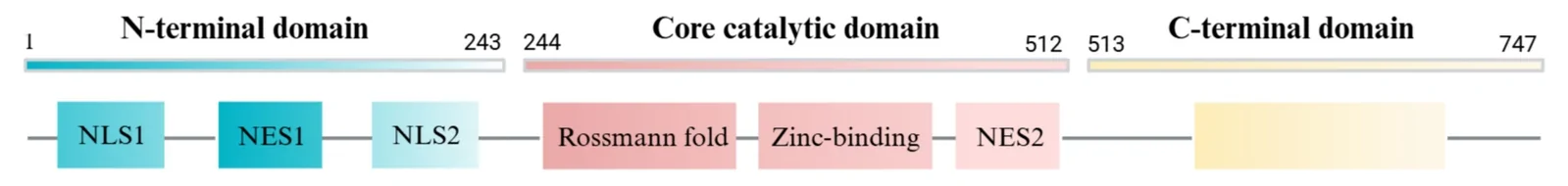
**Domain architecture of the human SIRT1 protein**. The primary structural domains of the SIRT1 protein include an N-terminal regulatory domain, a core catalytic domain, and a C-terminal regulatory domain, encompassing a Rossmann fold subdomain, a zinc-binding subdomain, two NLSs, and two NESs.

SIRT1 is predominantly localized within the nucleus, where it directly catalyzes the deacetylation of numerous nuclear substrates, such as histones and critical transcription factors [19]. This nuclear compartmentalization is critical for its regulatory roles in epigenetic and transcriptional control [20]. Furthermore, the NLSs and NESs within the SIRT1 protein sequence mediate its dynamic nucleocytoplasmic shuttling [21]. This nucleocytoplasmic trafficking is rigorously controlled by various post-translational modifications [22], including phosphorylation [18], SUMOylation [23], ubiquitination [24], and redox modifications [25]. By modulating the stability, enzymatic activity, and subcellular distribution of SIRT1, these modifications collectively ensure the precise regulation of its cellular localization.

### 2.2 Biological functions and expression regulation of SIRT1

SIRT1 functions as a key sensor of the cellular metabolic status, playing a central role in regulating cardiac aging and maintaining metabolic homeostasis [26]. In the nucleus, SIRT1 catalyzes the deacetylation of histone residues such as H3K9, H3K14, and H4K16, facilitating heterochromatin formation and silencing senescence-associated and proapoptotic genes, thereby preserving genomic stability in cardiomyocytes [27]. In energy metabolism, SIRT1 acts through multitiered mechanisms: it promotes mitochondrial biogenesis and oxidative phosphorylation by deacetylating and activating peroxisome proliferator-activated receptor gamma coactivator 1-alpha (PGC-1α) [28], while concurrently enhancing fatty acid oxidation (FAO) via cooperative activation of AMP-activated protein kinase (AMPK) and peroxisome proliferator-activated receptor alpha (PPARα), thus securing a continuous energy supply [29]. In addition to metabolic regulation, SIRT1 deacetylates the tumor protein p53 (p53) to suppress apoptosis [30] and enhances forkhead box protein O1/O3 (FOXO1/3) transcriptional activity, increasing the expression of antioxidant enzymes and autophagy-related genes to facilitate ROS clearance and maintain cellular homeostasis [31-33]. Experimental studies have confirmed that moderate SIRT1 overexpression mitigates age-related cardiac hypertrophy, fibrosis, and functional decline [34, 35]. In pathological settings such as ischemia/reperfusion (I/R) injury, SIRT1 exerts multifaceted protection—suppressing NF-κB-mediated inflammatory cytokine release and activating endothelial nitric oxide synthase (eNOS) to improve myocardial perfusion [36, 37]. Collectively, these multidimensional mechanisms establish SIRT1 as a pivotal therapeutic target for sustaining cardiac homeostasis and countering aging-related cardiovascular pathologies (Fig 2).

**Figure 2.**
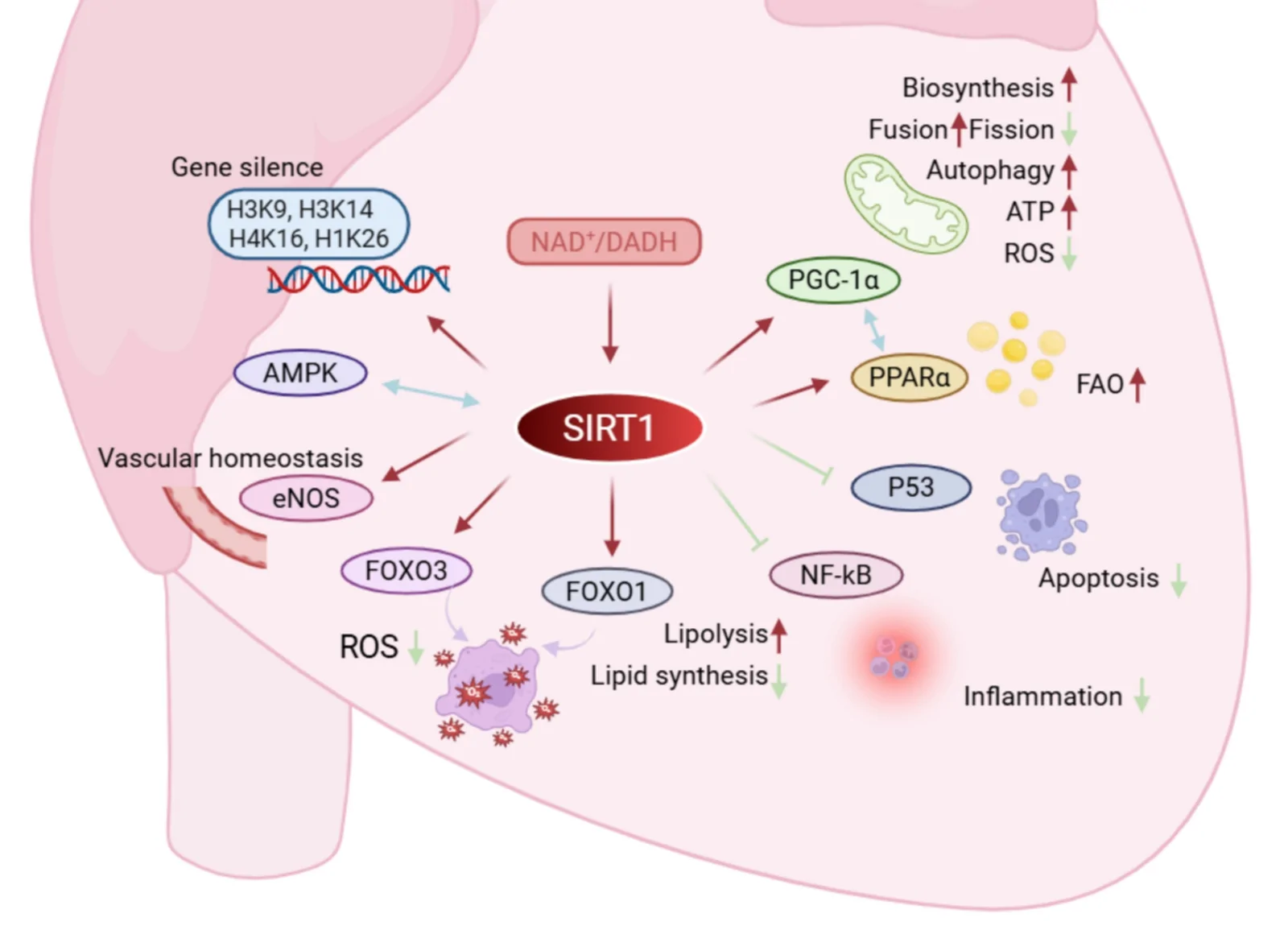
**Multifunctional roles of SIRT1 in cardioprotection**. SIRT1 represses the transcription of senescence-associated genes by deacetylating lysine residues at histone sites, including H3K9, H3K14, H4K16, and H1K26. It activates PGC-1α to regulate mitochondrial function and enhances FAO through the activation of AMPK and PPARα. SIRT1 suppresses apoptosis by deacetylating p53, inhibits NF-κB signaling to attenuate inflammation, and promotes ROS scavenging through the deacetylation of FOXO1/3. Additionally, SIRT1 activates eNOS to improve myocardial perfusion.

The expression and activity of Sirtuin 1 (SIRT1) are precisely regulated through multiple layers of control mechanisms. Epigenetically, DNA hypermethylation of the SIRT1 gene promoter suppresses transcription and reduces mRNA abundance by blocking transcription factor binding [38]. Transcriptionally, factors such as c-MYC, E2F transcription factor 1 (E2F1), and FOXO3a positively regulate SIRT1 expression [39-41], whereas p53 and hypermethylated in cancer 1 (HIC1) function as transcriptional repressors [42, 43]. Posttranscriptional control is mediated by microRNAs (miRNAs), including miR-34a, miR-9, and miR-217, which bind the 3'-untranslated region of SIRT1 mRNA to induce degradation or translational repression [44]. At the posttranslational level, SIRT1 protein stability is maintained by a dynamic balance between ubiquitination and deubiquitination. E3 ubiquitin ligases, such as SMAD specific E3 ubiquitin protein ligase 2 (SMURF2), mouse double minute 2 homolog (MDM2), the anaphase-promoting complex/cyclosome (APC/C), and the cofactor ubiquitin conjugating enzyme E2 V1 (Ube2v1), mediate polyubiquitination and subsequent degradation of SIRT1 [45-48]. Conversely, deubiquitinating enzymes including ubiquitin specific peptidase 22 (USP22) and ubiquitin specific peptidase 4 (USP4), enhance SIRT1 stability by removing ubiquitin chains [49, 50]. Furthermore, SIRT1 deacetylase activity is directly modulated by protein-protein interactions: binding with the cell cycle and apoptosis regulator 2 (CCAR2), deleted in breast cancer 1 (DBC1), and phosphofurin acidic cluster sorting protein 2 (PACS-2) inhibits its activity [51-53], whereas active regulators of SIRT1 (AROS) and AMP-activated protein kinase (AMPK) serve as positive regulators [54, 55]. Collectively, these interconnected regulatory mechanisms ensure the precise control and functional adaptability of SIRT1 (Fig. 3).

**Figure 3.**
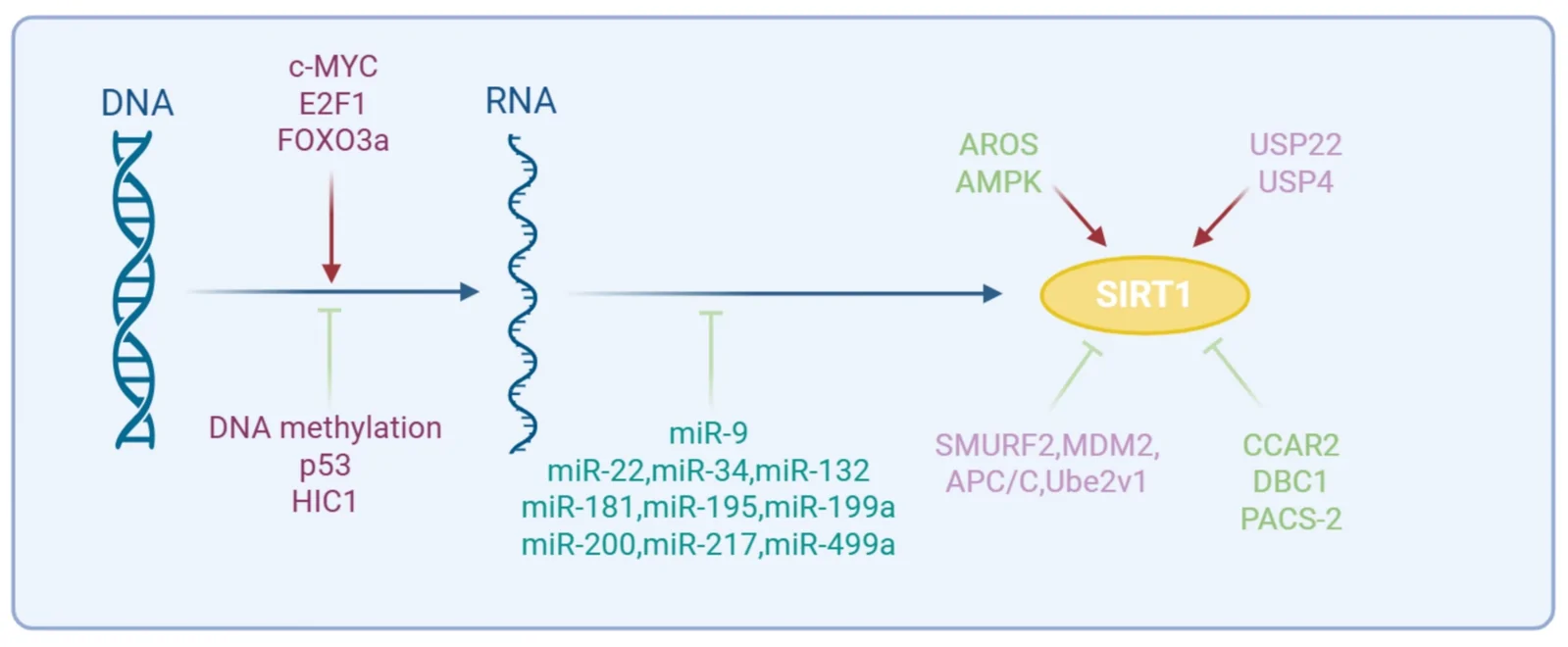
**Multilevel regulation of SIRT1 expression and activity**. Epigenetic regulation: Transcriptional repression of the SIRT1 gene via DNA hypermethylation. Transcriptional regulation: SIRT1 transcription is promoted by c-MYC, E2F1, and FOXO3a but suppressed by p53 and HIC1. Posttranscriptional regulation: miR-9, miR-22, miR-34a, miR-132, miR-181, miR-195, miR-199a, miR-200, miR-217, and miR-449a inhibit SIRT1 expression. Ubiquitination: SMURF2, MDM2, APC/C, and Ube2v1 mediate SIRT1 ubiquitination and promote its proteasomal degradation, whereas the deubiquitinating enzymes USP22 and USP4 increase SIRT1 stability. Protein interaction networks: CCAR2, DBC1, and PACS-2 inhibit SIRT1 deacetylase activity through direct binding, whereas AROS and AMPK increase its activity

## 3. Role of SIRT1 in cardiac aging

### 3.1 Changes in SIRT1 expression in aging hearts

SIRT1 expression and activity progressively decrease with the progression of cardiac aging and related diseases [56]. Clinical observations have revealed that myocardial tissue from patients with advanced heart failure shows significantly reduced SIRT1 protein expression [57]. Similarly, the levels of SIRT1 mRNA and protein in circulating endothelial progenitor cells from patients with coronary artery disease are markedly lower than those in healthy control subjects [58]. Basic research has elucidated multiple cardioprotective mechanisms of SIRT1. Genetic studies have demonstrated that cardiomyocyte-specific knockout of Sirt1 induces cardiac dysfunction in mice, confirming its essential role in maintaining normal heart function [59]. At the molecular level, SIRT1 activates the AMPK/PGC-1α pathway, thereby inhibiting pressure overload-induced cardiac hypertrophy [29]. Additionally, SIRT1 suppresses inflammation and oxidative stress by modulating nuclear factor kappa-light-chain-enhancer of activated B cells (NF-κB) signaling [60]. In regulating cell death, SIRT1 inhibits pyroptosis and enhances autophagic flux, ameliorating cardiac damage induced by sepsis or specific drugs [61, 62]. From a metabolic perspective, the adipokine Isthmin-1 prevents aging-related cardiac dysfunction by enhancing glycolysis and SIRT1 activity [34], while direct supplementation with recombinant SIRT1 protein improves myocardial lipid metabolism and diastolic function in db/db mice [63]. Clinical interventions, including nonpharmacological approaches such as intermittent hypoxia/ischemia training and caloric restriction, and pharmacological agents such as resveratrol, omega-3 fatty acids combined with vitamin E, and phosphodiesterase 5 inhibitors, effectively upregulate SIRT1 expression or activity, leading to improved metabolic status and cardiovascular function [64-68]. In summary, these findings establish a solid experimental and theoretical foundation for targeting SIRT1 to intervene in cardiac aging and associated pathologies.

### 3.2 SIRT1 as a regulatory hub in age-related cardiac pathogenesis

Cardiac aging involves progressive mitochondrial deterioration, characterized by structural alterations such as volume loss and crista disassembly, along with mtDNA mutation accumulation, ultimately disrupting mitochondrial homeostasis [69]. Given this central role of mitochondrial dysfunction, therapeutic strategies aimed at modulating SIRT1 activity have emerged as promising approaches to retard cardiac aging. Experimental evidence indicates that SIRT1 activation rescues heart failure in SIRT3-knockout mice by improving mitochondrial function and ATP production [70]. In I/R injury models, SIRT1 confers protection through increased mtDNA copy number, elevated ATP synthesis efficiency, and reduced ROS levels—effects abolished by SIRT1 knockout [71]. Moreover, SIRT1 activation mitigates myocardial infarction damage following miR-30a-5p depletion, restoring the mitochondrial membrane potential and respiratory chain complex activity [72]. In high-fat diet-induced diabetic cardiomyopathy, SIRT1 retards pathological progression by suppressing the release of mtDNA-enriched vesicles and inhibiting opening of the mitochondrial permeability transition pore (mPTP) [73]. Thus, by orchestrating mitochondrial homeostasis through multifaceted mechanisms, SIRT1 represents a pivotal target for therapeutic intervention in cardiac aging and its associated pathologies.

**Figure 4.**
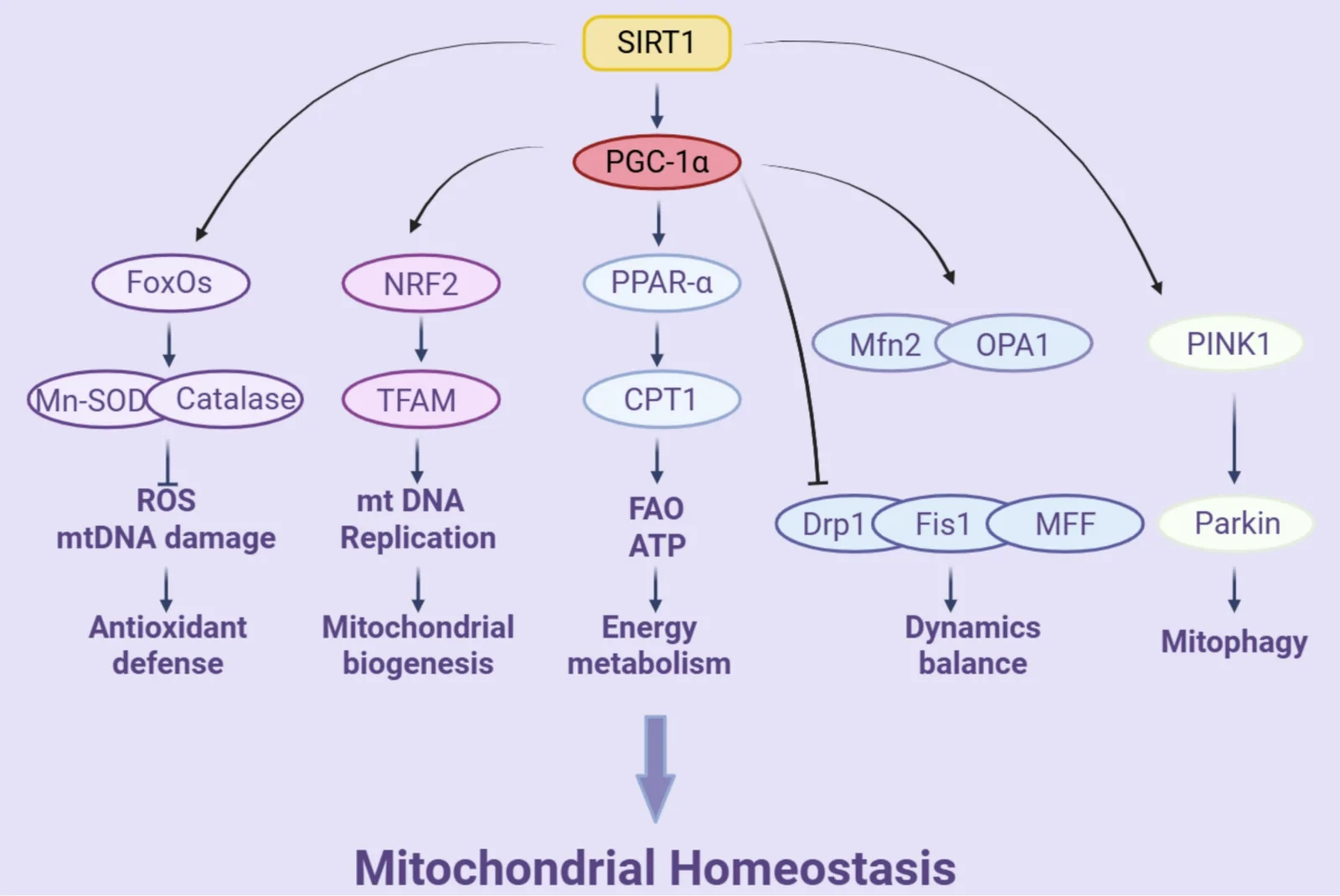
**Core mechanisms of mitochondrial homeostasis regulated by SIRT1**. SIRT1 orchestrates mitochondrial homeostasis by regulating mitochondrial biogenesis, antioxidant defense, energy metabolism, dynamic balance, and mitophagy.

## 4. SIRT1-mediated mitochondrial homeostasis in cardiac aging

SIRT1 is characterized by its ability to directly perceive the cellular energy status. During energy deprivation or mitochondrial impairment, SIRT1 activation triggers the coordination of downstream transcription factors and signaling pathways to regulate cellular processes, including mitochondrial biogenesis, the oxidative stress response, energy metabolism, dynamic equilibrium, and autophagy (Figure 4). Rather than acting as an isolated “master regulator”, SIRT1 functions as a pivotal hub for signal integration. For example, in mitochondrial biogenesis, SIRT1 cooperates with AMPK to activate PGC-1α [74]. In mitigating oxidative stress, SIRT1 regulates FOXO transcription factors, thereby complementing pathways such as those mediated by NRF2 [22]. Within the broader framework of mitochondrial regulation, nuclear-localized SIRT1 and SIRT3 play distinct yet complementary roles [75]. This network-based operational mode not only ensures the robustness of cellular homeostasis but also underscores the centrality of SIRT1 in integrating multifaceted signals to achieve precise regulatory outcomes.

### 4.1 Regulation of mitochondrial biogenesis

Mitochondrial biogenesis is the regulated cellular process by which new mitochondria are generated through the replication of mitochondrial DNA, synthesis of proteins, and assembly of membrane structures [76]. An age-related decline in this process is considered a central pathological mechanism in cardiac aging. SIRT1 serves as a critical regulator by activating the transcriptional coactivator PGC-1α. Once activated, PGC-1α stimulates the expression of downstream genes, such as NRF1/2 and mitochondrial transcription factor A (TFAM) [77, 78]. This cascade promotes mitochondrial DNA replication and the synthesis of respiratory chain proteins. In the aging heart, the expression or activity of SIRT1 is frequently diminished, leading to persistent hyperacetylation and functional inhibition of PGC-1α [74]. Suppression of the SIRT1-PGC-1α axis thereby impairs mitochondrial biogenesis, resulting in a reduced mitochondrial population and functional decline [79]. Experimental studies have demonstrated that cardiac-specific overexpression of SIRT1 increases mitochondrial DNA copy number and enhances the activity of respiratory chain complexes in aged mice [80]. Similarly, in progeroid mouse models with lamin A/C deficiency, SIRT1 activation ameliorates cardiac dysfunction and delays aging phenotypes [81]. Furthermore, in a LARP7 cardiomyocyte-specific knockout model, reduced SIRT1 expression and activity impaired mitochondrial biogenesis and oxidative phosphorylation (OXPHOS), which was reversed with a SIRT1 activator [82]. In summary, enhancing the SIRT1-PGC-1α signaling pathway promotes mitochondrial biogenesis and represents a promising strategy for mitigating cardiac aging (Fig. 5).

**Figure 5.**
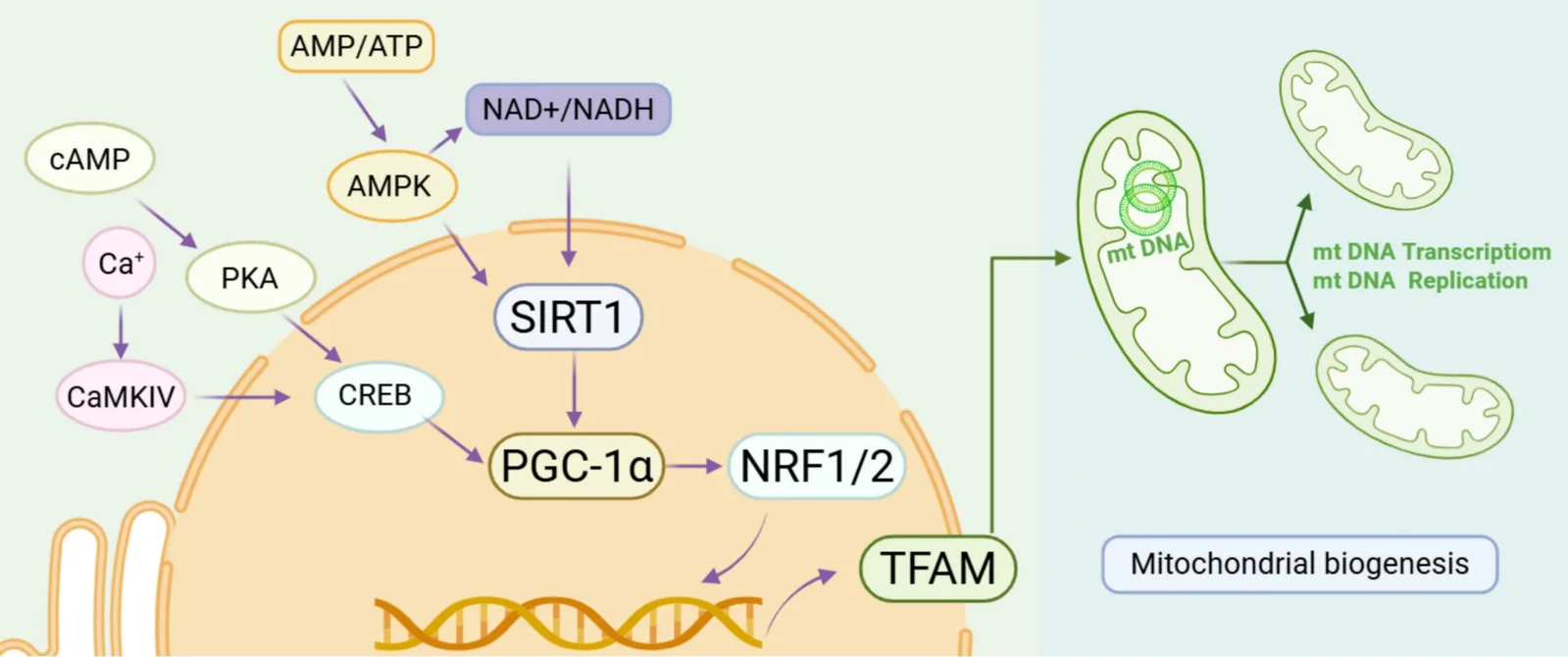
**SIRT1 promotes mitochondrial biogenesis**. SIRT1 deacetylates and activates PGC-1α, which subsequently upregulates the expression of NRF1/2 and TFAM, thereby initiating mtDNA replication and synthesizing the mitochondrial proteins essential for the generation of new mitochondria.

### 4.2 Enhancement of mitochondrial antioxidant defense

The free radical theory of aging proposed by Harman (1956) and further refined by Miquel and colleagues (1980) suggests that the accumulation of oxidative damage is a primary driver of the aging process [83]. In the aging heart, diminished function of the mitochondrial electron transport chain (ETC), particularly in complexes I and III, increases electron leakage and the excessive production of ROS, including superoxide anion (O_2_•^-^) and hydrogen peroxide (H_2_O_2_) [84, 85]. This ROS surge induces irreversible mitochondrial damage through lipid peroxidation, protein modification, and DNA damage [86]. A concurrently compromised endogenous antioxidant defense system, marked by reduced activity of enzymes such as Cu/Zn-superoxide dismutase (Cu/Zn-SOD), Mn-SOD, and glutathione peroxidase (GPx), further exacerbates intramitochondrial oxidative stress [87-89]. The vulnerability of mtDNA to oxidative attack is heightened by its proximity to the ETC and lack of histone protection^23^. Consequently, the interplay of rampant ROS production, weakened antioxidant defenses, and accumulated mtDNA damage establishes a vicious cycle termed “ROS-induced ROS release” (RIRR). This cycle represents a core pathological mechanism driving the persistent exacerbation of oxidative stress in the aging heart [90, 91].

**Figure 6.**
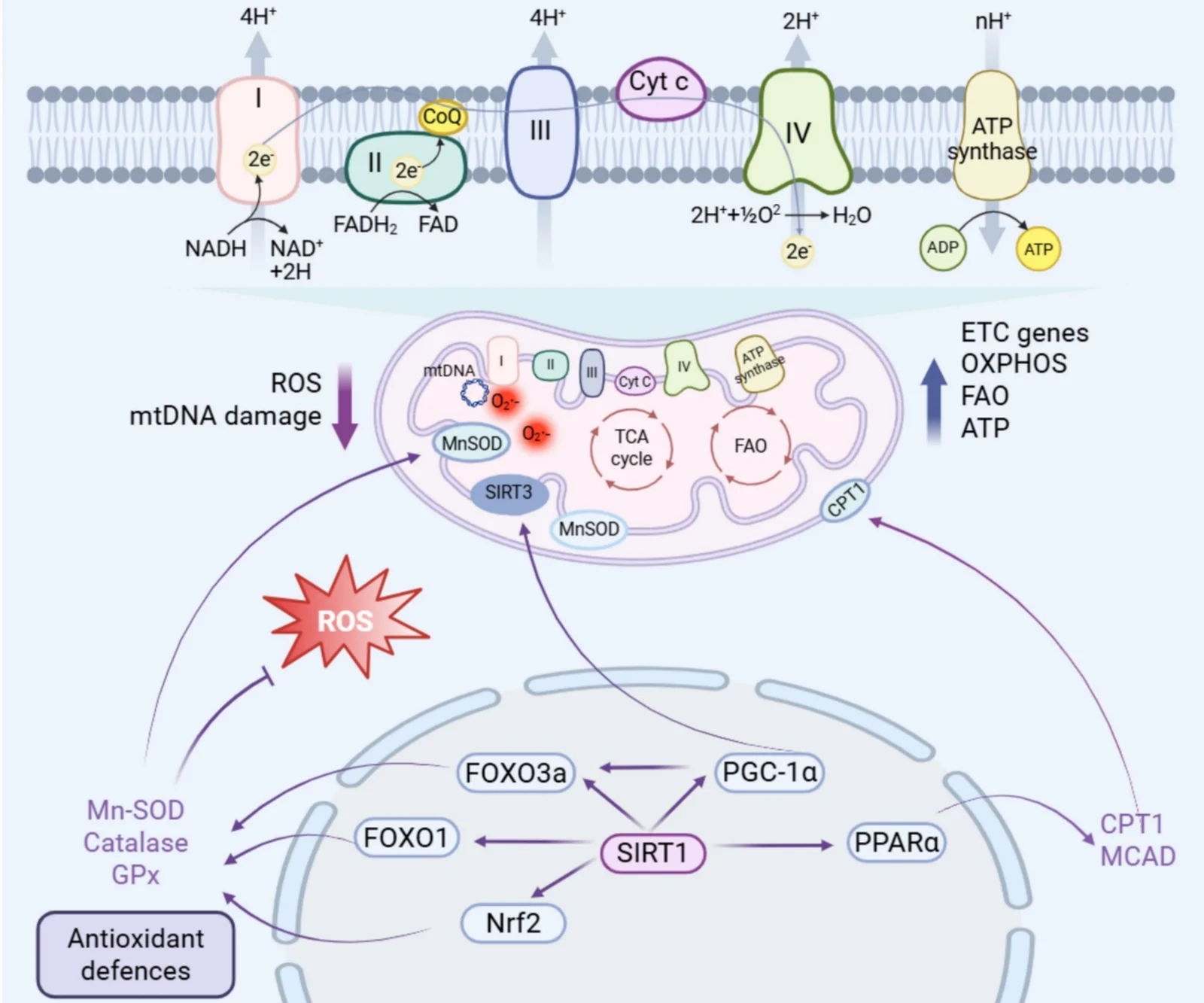
**SIRT1 enhances mitochondrial antioxidant defense and regulates energy metabolism**. SIRT1 delays cardiac aging through coordinated regulation of multiple signaling pathways. In terms of antioxidant defense, SIRT1 deacetylates the transcription factors FOXO1, FOXO3, and NRF2, increasing their transcriptional activity and nuclear stability and thereby promoting the expression of key antioxidant enzymes, including Mn-SOD, catalase, and GPx. This effectively reduces ROS levels and alleviates oxidative damage. With respect to energy metabolism, SIRT1 deacetylates and activates PGC-1α, strengthening its transcriptional synergy with PPARα and increasing the expression of rate-limiting enzymes in the FAO pathway, such as CPT1 and MCAD. Consequently, mitochondrial fatty acid metabolism is improved, and ATP synthesis efficiency in aged cardiomyocytes is restored. Additionally, activated PGC-1α induces the expression of the mitochondrial deacetylase SIRT3, which further attenuates mitochondrial oxidative stress and mtDNA damage, thereby reinforcing antioxidant defense. SIRT1 also indirectly facilitates the expression and assembly of electron transport chain components via coactivators such as PGC-1α, supporting efficient OXPHOS and collectively maintaining mitochondrial homeostasis. These integrated mechanisms ultimately contribute to the synergistic antiaging effects of SIRT1 in the heart.

SIRT1 confers cardioprotection in various cardiac pathology models by mitigating mitochondrial oxidative stress. In cardiac I/R injury, it enhances antioxidant defense through deacetylation of the transcription factor FOXO1, which upregulates Mn-SOD [92]. Similarly, in isoproterenol-induced hypertensive cardiac remodeling, SIRT1 improves pathological remodeling by driving antioxidant gene expression via FOXO3a [93]. This action is amplified by the key coactivator PGC-1α, which acts synergistically with SIRT1 on FOXO3a to promote a broad antioxidant response, including Mn-SOD and catalase, forming a positive feedback loop [94]. SIRT1 also functions cooperatively with SIRT3 to maintain mitochondrial homeostasis; an age-related deficiency in both sirtuins significantly impairs mitochondrial ROS clearance, thereby exacerbating cardiomyocyte contractile dysfunction [11]. Taken together, these mechanisms identify targeted SIRT1 activation as a promising strategy to counteract cardiac aging and associated oxidative damage (Fig. 6).

### 4.3 Modulation of energy metabolism

Impaired energy metabolism is a central driver of the progressive functional decline in the aging heart, characterized by remodeling of substrate utilization and reduced mitochondrial bioenergetic efficiency [95]. In healthy adults, the heart derives approximately 95% of its ATP from mitochondrial OXPHOS, with FAO serving as the primary energy source [6]. However, the aging heart undergoes metabolic reprogramming, shifting from predominantly fatty acid utilization to increased glycolysis [96]. This shift coincides with a reduced capacity to oxidize fatty acids and ketone bodies [97], alongside altered insulin signaling [98], impairing energy reserves and metabolic flexibility [99]. In murine models, aging hearts exhibit diminished ETC activity, a decrease in the mitochondrial membrane potential (ΔΨm), and significant reductions in ATP synthesis [100, 101]. Consistent with these findings in animal models, the phosphocreatine/ATP ratio is significantly lower in the aging human female heart, reflecting a decline in cardiac energy reserve [102]. Furthermore, age-related erosion of endoplasmic reticulum-mitochondria contacts disrupt lipid metabolism, exacerbating FAO dysfunction [103]. Together, these defects contribute to an “energy crisis” in the senescent heart.

SIRT1 ameliorates the decline in cardiac function during aging by regulating mitochondrial energy substrate homeostasis. It acts by deacetylating PGC-1α, which enhances PGC-1α/PPARα synergy to upregulate FAO enzymes, including carnitine palmitoyltransferase 1 (CPT1) and medium-chain acyl-CoA dehydrogenase (MCAD), thereby restoring ATP synthesis efficiency in the aging myocardium [104]. Consistent with this role, activation of the SIRT1/PGC-1α axis in diabetic cardiomyopathy models improves mitochondrial fatty acid oxidation and reduces lipid deposition [105-107]. Conversely, cardiomyocyte-specific SIRT1 knockout impairs AMPK phosphorylation, attenuating the inhibitory phosphorylation of acetyl-CoA carboxylase (ACC); the resulting accumulation of malonyl-CoA inhibits CPT1-mediated fatty acid transport into mitochondria, precipitating a myocardial energy crisis [108]. Therefore, targeting the SIRT1-mediated regulation of mitochondrial energy metabolism represents a promising therapeutic strategy for cardiac aging (Figure 6).

### 4.4 Maintenance of mitochondrial dynamics balance

Mitochondrial dynamics are maintained by an equilibrium between fission and fusion, processes governed by a suite of highly conserved proteins [109]. Fusion is mediated by outer-membrane mitofusins (Mfn1/2) and optic atrophy 1 (OPA1), whereas fission is driven by cytosolic dynamin-related protein 1 (Drp1) upon its recruitment to mitochondria [110]. In the aging heart, this balance is often disrupted, manifesting as excessive fission and suppressed fusion, which leads to mitochondrial fragmentation, loss of membrane potential, and functional impairment [111]. SIRT1 is a pivotal regulator of this balance. By suppressing excessive mitochondrial fission, SIRT1 alleviates cardiac dysfunction under high-altitude hypoxia via the PGC-1α-DRP1/FIS1/MFF axis [112], and protects against myocardial injury in hemorrhagic shock by activating the SIRT1/AMPK/Drp1 pathway [113]. In promoting fusion, the SIRT1 activator nicotinamide riboside (NR) upregulates Mfn2 expression through the SIRT1-PGC-1α-PPARα signaling cascade, thereby enhancing mitochondrial network connectivity [114]; concurrently, resveratrol coactivates SIRT1 and SIRT3, not only boosting Mfn2 and OPA1 expression but also restoring the mitochondrial quality control network postischemia-reperfusion injury via augmented Parkin-mediated mitophagy [115]. Furthermore, the activation of FMS-like tyrosine kinase 3 stabilizes the fusion protein OPA1 and reduces mitochondrial fragmentation by inhibiting p53 acetylation—a mechanism intrinsically linked to the deacetylase activity of SIRT1 [116, 117] (Fig. 7).

### 4.5 Promotion of mitophagy

Mitophagy, the selective autophagic degradation of mitochondria, is essential for cardiomyocyte health through the targeted clearance of dysfunctional organelles [118, 119]. Under physiological conditions, mitochondrial damage triggers the stabilization of PTEN-induced kinase 1 (PINK1) on the outer mitochondrial membrane, which recruits and activates Parkin; activated Parkin subsequently ubiquitinates mitochondrial proteins and recruits autophagy receptors such as optineurin (OPTN) and nuclear dot protein 52 (NDP52), thereby initiating mitophagy [120, 121]. However, in aging cardiomyocytes, reduced PINK1 stability and impaired Parkin activity compromise the efficiency of mitochondrial clearance [122]. Additionally, dysregulation of noncanonical mitophagy pathways mediated by BCL2-interacting protein 3 (BNIP3), BCL2 interacting protein 3 like (BNIP3L/NIX), and FUN14 domain containing 1 (FUNDC1) further disrupts mitochondrial quality control and energy homeostasis [123]. During aging, the dysregulation of mitophagy is closely associated with imbalances in multiple signaling pathways, including suppressed insulin-like growth factor 1 receptor (IGF-1R) signaling^143^, decreased growth differentiation factor 11 (GDF11) expression^144^, diminished PGC-1α activity [124], hyperactivation of the Toll-like receptor 4 (TLR4)-mediated inflammatory pathway [125], and decreased Nrf2-dependent antioxidant function [126, 127]. Overall, these abnormalities foster an inhibitory cellular microenvironment, leading to a sustained decline in mitophagic flux and ultimately contributing to cardiomyocyte dysfunction.

SIRT1 plays a critical role in regulating mitophagy within the aging myocardium, largely through its modulation of the canonical PINK1/Parkin pathway [128, 129]. This regulatory function is further supported by brain and muscle ARNT-Like 1 (BMAL1), which enhances mitophagic flux via positive regulation of SIRT1 signaling, thereby attenuating sepsis-induced myocardial injury [130]. Additionally, activation of the SIRT1-FOXO1 axis markedly upregulates key autophagy-related genes, facilitating the efficient recognition and clearance of damaged mitochondria [131]. In H9C2 cells subjected to I/R injury, downregulation of miR-217-5p promotes mitophagy and confers cardioprotection by reversing its inhibitory effect on SIRT1 activity [132] (Fig. 7).

**Figure 7.**
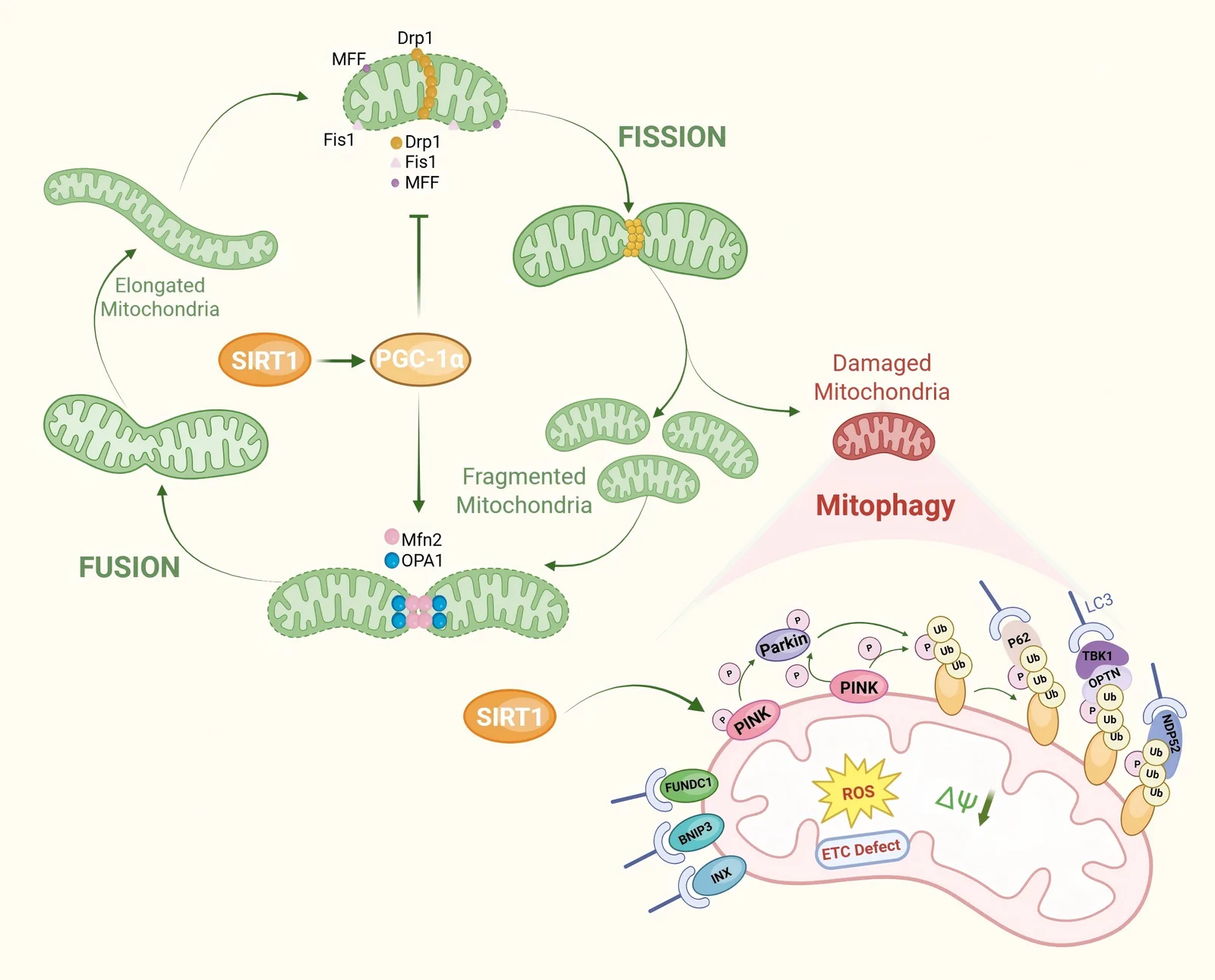
**SIRT1 regulates mitochondrial dynamics and enhances mitophagy**. SIRT1 activates the PGC-1α signaling pathway; downregulates the expression of the mitochondrial fission proteins DRP1, FIS1, and MFF to suppress excessive mitochondrial fission; and concurrently promotes the expression of the fusion proteins MFN2 and OPA1 to increase the mitochondrial fusion capacity, thereby maintaining the homeostasis of mitochondrial dynamics. In parallel, SIRT1 augments the PINK1/Parkin-mediated mitophagy pathway, facilitating the recognition and clearance of damaged mitochondria and preserving mitochondrial quality control, ultimately alleviating the process of cardiac aging.

## 5. Therapeutic strategies targeting SIRT1 for cardiac aging

Therapeutic strategies targeting SIRT1 have recently attracted interest for mitigating cardiac aging. Accumulating evidence has demonstrated that SIRT1-positive modulators alleviate cardiac aging and related pathological phenotypes by preserving mitochondrial homeostasis and counteracting mitochondrial dysfunction (Table 1). Furthermore, translational studies have provided preliminary validation of the potential to target SIRT1 to improve cardiac function in clinical settings (Table 2).

**Table 1:** Positive modulators of SIRT1 as potential therapies.

Drugs	Experimental model	Molecular mechanism	Outcomes	Limitations	Ref.
**Ginsenoside Rc**	I/R-H9c2, mice	Enhances mitochondrial biogenesis via the SIRT1-PGC-1α-NRF1 axis, boosting oxidative phosphorylation and ATP synthesis	Activate the SIRT1-PGC1α pathway to enhance glucose metabolism and mitochondrial function	The oral bioavailability has not been adequately assessed	[133]
**Tanshinone IIA**	I/R- CMECs, mice	Activate the SIRT1/PGC-1α pathway, sustaining mitochondrial potential, reducing pro-apoptotic factor leakage, and inhibiting mitochondrial apoptosis	Attenuate microvascular wall destruction, lumen stenosis, and CMEC apoptosis, improve microvascular homeostasis and cell survival	Focus on microvascular endothelium; direct cardiomyocyte effects remain incompletely elucidated; translational potential requires validation	[134]
**Bakuchiol**	I/R- NRCMs, rats	Activate SIRT1/PGC-1 to enhance mitochondrial biogenesis and antioxidant capacity, and inhibit apoptosis	Attenuate myocardial I/R injury, reduce infarct size, and improve mitochondrial function	Incomplete pharmacokinetic data; preliminary evidence	[135]
**Berberine**	Aged rats, DOX-H9c2	Regulate the Klotho/SIRT1 signaling pathway, mitigating cellular senescence	Attenuate cardiac senescence, improve cardiac function	Preclinical models; potentially oversimplified mechanism; incomplete causal evidence; unconfirmed human relevance	[136]
Melatonin	DM-mice, HG-H9c2	Activate the SIRT1/PGC-1α pathway, leading to inhibition of Drp1-mediated mitochondrial fission, reducing oxidative stress and cardiomyocyte apoptosis	Prevent diabetes-induced cardiac dysfunction	Unverified translational potential from preclinical animal models to human diseases	[137]
	**DXR-rats, H9c2**	Increase SIRT1 and PGC-1α expression, improve mitochondrial function, and reduce mitochondrial fission	Improve cardiac output in DXR-induced cardiotoxicity, reduce tumor growth, and decrease cardiomyocyte cell death	Acute cardiotoxicity model; mechanistic insights into PGC-1α/SIRT1 not fully elucidated; pleiotropic effects of melatonin	[138]
**Apigenin**	Dox-mice, HL-1	Activate the Sirt1/Atf5 pathway to regulate mitochondrial unfolded protein response	Restore heart function, reduce myocardial swelling, and inhibit heart inflammation	Acute model limitation; incomplete pharmacokinetic data	[139]
**Quercetin**	SIC-rats, H9C2	Activate the SIRT1/p53/SLC7A11 signaling pathway, inhibiting ferroptosis in cardiomyocytes	Ameliorate SIC, improve cardiac function	Sepsis model complexity	[140]
**SRT1720**	I/R-mice	SIRT1 modulates cardiac NLRP3 inflammasome through pyruvate dehydrogenase	Improve cardiac systolic function, reduce infarct size, and cardiomyocyte pyroptosis	Lack of long-term safety and efficacy data in large animals or humans	[141]
**NMN**	Sirt3^-/-^mice	Activate the SIRT1/PGC-1α pathway, improve mitochondrial biosynthesis and oxidative respiration	Improve cardiac function	Limited clinical translatability	[70]

### 5.1 Natural product activators

Natural products, representing a rich source of therapeutic candidates, have been reported to directly or indirectly modulate SIRT1 to provide cardioprotection against cardiac aging [133]. The polyphenol resveratrol is one of the most thoroughly investigated SIRT1 activators. Resveratrol activates the SIRT1/PGC-1α pathway, thereby enhancing mitochondrial biogenesis and promoting autophagy [134]. It also upregulates eNOS-SIRT1 expression in diabetic cardiomyocytes, which improves mitochondrial energy metabolism [135], and suppresses apoptosis via SIRT1-mediated deacetylation of p53 [136, 137]. However, its therapeutic efficacy is limited by poor bioavailability [138]. Furthermore, high-dose administration has been associated with a risk of adverse effects [139]. In addition to resveratrol, other natural compounds, including ginsenoside Rc, tanshinone IIA, curcumin, and bakuchiol, also enhance mitochondrial function in cardiomyocytes by activating SIRT1, although their utility is similarly hampered by low bioavailability [140-144]. Additionally, melatonin inhibits Drp1-mediated mitochondrial fission in diabetic hearts via the SIRT1/PGC-1α pathway [145], and ameliorates mitochondrial function in models of doxorubicin-induced cardiotoxicity [146]; its antiaging effects have also been linked to the suppression of the NLRP3 inflammasome [147]. Apigenin modulates the mitochondrial unfolded protein response through the Sirt1/Atf5 pathway, improving doxorubicin-induced cardiac dysfunction [148]. Quercetin alleviates sepsis-induced cardiomyopathy by activating SIRT1 and exerting antioxidant effects [149]. It should be noted, however, that the cardioprotective properties of these natural products are largely derived from cardiotoxicity models, with evidence in pure aging models remaining limited. Therefore, targeting the SIRT1-mitochondrial axis via natural products represents a promising but still exploratory approach for intervening in cardiac aging and related pathologies.

**Table 2:** Comparison between preclinical studies and clinical trials of SIRT1-targeted therapeutic strategies.

Drugs	Type	Experimental model/ Study Population	mechanism	Results	Limitations	Ref.
Resveratrol	Preclinical experiment	T2DM- rats	Activate SIRT1/PGC-1α, improve mitochondrial biosynthesis, enhance antioxidant defense and energy metabolism	Improve cardiac function, reduce myocardial hypertrophy, and alleviate the disorder of myocardial fiber arrangement	Lack of long-term safety data; clinical translation requires validation of human-equivalent dosing and long-term efficacy	[143]
		**I/R-T2DM rats**	Enhance eNOS-SIRT1 expression, promote high-energy compound production	Alleviate I/R injury, improve diastolic function, enhance energy metabolism	The exclusive use of female rats presents a limitation regarding biological sex; the mechanistic insights into the eNOS-SIRT1 pathway, including its upstream regulators and downstream effectors, require further clarification	[144]
		**Dox- mice**	SIRT1 deacetylates p53 to suppress its transcriptional activity, reducing cardiomyocyte apoptosis.	Alleviate Dox-induced cardiomyocyte apoptosis and cardiotoxicity	The cell specificity of the SIRT1-p53 pathway requires further validation; the Dox injury model differs from the mechanisms of metabolic cardiomyopathy	[145]
		**A/R- NRCMs**	Activate SIRT1 to deacetylate VDAC1, reducing mitochondria-mediated apoptosis	Reduce A/R-induced cardiomyocyte apoptosis and protect cardiac function	This study only uses *in vitro* cell models and lacks in vivo validation	[146]
	**Clinical trial**	T2DM and CHD subjects	Upregulate SIRT1 and PPAR-γ expression in PBMCs, reduce oxidative stress, improve insulin signaling	Improve blood sugar and lipid levels	Short Duration: 4-week intervention; Limited Population: Focused on T2DM+CHD patients; Mechanistic Depth: SIRT1 upregulation was measured in PBMCs, limiting direct mechanistic claims for heart-specific effects; Dose Exploration: Single dose used	[68]
SRT2104	Preclinical experiment	TGF-β-H5V cells	Activate SIRT1, reduce TGF-β receptor 1 and phosphorylated Smad2/3 expression, inhibited Smad2/3 nuclear translocation	Attenuate cardiac fibrosis	Mechanistic depth: the precise molecular details of how SIRT1 activation leads to Smad2/3 inhibition warrant further investigation	[151]
	Clinical trial	healthy cigarette smokers and T2DM subjects	SIRT1 activation may improve vascular function by enhancing the bioavailability of nitric oxide and inhibiting oxidative stress	Reduce TC and LDL-C levels, reduce PAI-1 levels	Improve augmentation pressure and augmentation index	[152]
		**T2DM subjects**	Activate SIRT1, modulating pathways involved in endothelial function, fibrinolysis, and metabolism	Attenuate bradykinin-induced vasodilatation, reduce net PAI-1 antigen release, induce weight loss, but increase HbA1c	Small Sample Size; Inconsistent Results: Mix of neutral, beneficial, and adverse outcomes complicates interpretation	[153]
Nicotinamide Riboside	Preclinical experiment	DOX-hiPSC-CMs	Activate the SIRT1/ERRα signaling pathway	Improve mitochondrial function, attenuate DOX-induced cardiomyocyte apoptosis and contractile dysfunction	Findings are confined to an in vitro hiPSC-CM model	[154]
		**DOX-mice,PMVMs**	Enhance autolysosome clearance via the NAD^+^/SIRT1 pathway.	Improve cardiac function and reduce myocardial injury	The therapeutic window requires further investigation	[155]
	**Clinical trial**	Patients with heart failure with HFrEF	Increase NAD^+^ levels, improve mitochondrial function, inhibit NLRP3 inflammasome	NR is well tolerated in patients with HFrEF	Small sample size; The myocardial NAD^+^ level is not directly evaluated; NR metabolites are not quantified	[156]

### 5.2 Synthetic SIRT1-activating compounds

The development of highly selective and potent synthetic SIRT1 agonists is a pivotal strategy for advancing targeted therapies. Preclinical studies have demonstrated that SRT2104, a highly selective SIRT1 activator, ameliorates cardiac fibrosis by regulating the endothelial-to-mesenchymal transition through the transforming growth factor (TGF)-β1/Smad2/3 pathway [150]. Clinical investigations indicate that SRT2104 can modulate lipid profiles in healthy smokers [151]. In patients with type 2 diabetes, SRT2104 showed beneficial effects on arterial stiffness but did not significantly alter blood glucose or insulin levels [152, 153]; thus, its long-term efficacy and broader applicability necessitate validation in larger cohorts. Although short-term tolerance for this compound is favorable, its clinical utility may be constrained by limited oral bioavailability [154]. In preclinical studies, the synthetic agonist SRT1720 attenuated myocardial I/R injury by suppressing NLRP3 inflammasome activation via SIRT1-dependent regulation of pyruvate dehydrogenase [155]. Furthermore, certain anilino-pyridine derivatives exhibit cardioprotective effects at low concentrations [156]. These findings collectively endorse SIRT1 as a promising therapeutic target for cardiac aging and associated pathologies, while underscoring the persistent challenges in translating synthetic agonists to clinical practice.

### 5.3 NAD^+^ precursors

Supplementation with NAD^+^ precursors, including nicotinamide mononucleotide (NMN) and NR, indirectly enhances SIRT1 activity through the elevation of intracellular NAD^+^ levels [157]. Research demonstrates that NMN rescues cardiac function in Sirt3^-/-^ mice by activating the SIRT1/PGC-1α pathway to compensate for SIRT3 deficiency, thereby enhancing mitochondrial biogenesis and oxidative respiration [70]. Regarding NR, it attenuates doxorubicin-induced cardiotoxicity through the SIRT1/ERRα pathway [158] and helps maintain mitochondrial homeostasis in diabetic cardiomyopathy models via the SIRT1-PGC1α-PPARα axis [159]. Clinically, NR supplementation safely increases blood NAD^+^ levels in both heart failure patients and healthy volunteers [160, 161]. It also ameliorates the inflammatory status and mitochondrial dysfunction in the peripheral blood mononuclear cells (PBMCs) of heart failure patients [162]. Nevertheless, despite effectively elevating circulating NAD^+^, robust evidence demonstrating a direct improvement in cardiac function parameters from NR supplementation remains limited [163]. Consequently, large-scale, long-term clinical trials are imperative to conclusively determine the therapeutic benefits and potential risks of NAD^+^ precursor supplementation.

### 5.4 Gene-based Therapy

Gene therapy represents a novel strategy for restoring SIRT1 expression in the aging heart. Studies reveal that SIRT1 overexpression, delivered via cardiotropic adeno-associated virus serotype 9 (AAV9), ameliorates mitochondrial function, increases ATP production, and enhances resistance to ischemic stress in aged models [81, 108]. Additionally, the transplantation of SIRT1-modified mesenchymal stem cells has been shown to improve cardiac function in models of aging with myocardial infarction [164]. Despite the potential of these strategies, their clinical translation requires careful evaluation of associated risks, such as vector-induced immune responses and insertional mutagenesis.

## 6. Challenges in clinical translation

The clinical translation of SIRT1-targeted therapies for cardiac aging currently confronts several challenges. A primary issue is the low specificity and propensity for off-target effects associated with many SIRT1 modulators. Their mechanisms often involve cross-interference with multiple signaling pathways, exhibit poor tissue selectivity, and can consequently induce unintended physiological responses [165]. This problem involves high structural homology among sirtuin family members, which presents a significant technical barrier to developing highly selective SIRT1 agonists [166]. Another major challenge involves pharmacokinetic limitations. Natural activators such as resveratrol demonstrate low oral bioavailability; following a single oral dose of 25-150 mg, the maximum plasma concentration reaches only 1.48-24.8 ng/mL, which is insufficient to maintain therapeutic levels [167]. Although synthetic agonists such as SRT2104 are structurally optimized, their clinical efficacy remains suboptimal owing to unstable absorption and considerable interindividual variability, issues that are especially problematic in patients with heart failure [154, 168, 169]. The intrinsic limitations of SIRT1 modulators pose obstacles to their clinical translation. A core bottleneck hindering their clinical translation is the lack of reliable biomarkers. Although current studies suggest that SIRT1 activity in PBMCs and circulating miR-34a levels may serve as potential surrogate indicators, their ability to accurately reflect cardiac tissue status and inform clinical decision-making requires validation in large-scale clinical trials [58, 170, 171]. The absence of robust monitoring tools complicates the definition of an optimal dose and therapeutic window. The substantial gap between preclinical effective doses and human tolerable doses, the intrinsic circadian rhythm of SIRT1 activity, and its sensitive dose-response relationship collectively constitute a complex optimization problem [30, 172]. Moreover, existing research primarily consists of short-term phase I/II trials, resulting in a vacuum of long-term safety data—a critical concern for the long-term pharmacological management of chronic cardiac conditions [154, 168, 173]. In combination with these issues, growing evidence reveals the dual-edged nature of SIRT1 overactivation: sustained or excessive activation may not only directly impair cardiac function but also promote cancer progression, trigger metabolic disorders, or exacerbate inflammatory responses in other pathophysiological contexts [174-177]. Therefore, advancing SIRT1-targeted therapies from basic research to clinical application for cardiac aging requires overcoming multifaceted challenges, including precise drug design, pharmacokinetic optimization, establishment of clinical biomarkers, definition of the therapeutic window, and a deeper understanding of the dual biological roles of SIRT1.

## 7. Future Perspectives

Addressing the challenges in targeting SIRT1 to intervene in cardiac aging requires breakthroughs focused on the following directions. Future studies should prioritize elucidating the precise mechanisms by which SIRT1 regulates mitochondrial homeostasis, particularly the specific pathways operative in cardiac aging and the dynamic changes of key upstream and downstream regulatory molecules throughout the aging process. Another critical strategy involves developing highly selective allosteric activators of SIRT1 and optimizing drug formulations via novel technologies such as nanocarriers to improve targeting efficiency and safety [178-180]. For successful clinical translation, a multifaceted approach is essential. This includes the integration of multiomics data to identify composite biomarkers—such as mitochondrial DNA copy number, the NAD^+^/NADH ratio, and specific acetylation—to address the current lack of precise efficacy assessment tools. Furthermore, incorporating chronobiological investigations to understand the circadian rhythms of SIRT1 activity and cardiac function, alongside the adoption of adaptive clinical trial designs and pharmacokinetic/pharmacodynamic (PK/PD) modeling, will be instrumental in defining optimal therapeutic dosing and timing. Ultimately, establishing a comprehensive translational research pipeline, spanning from preclinical safety and efficacy studies to early-stage proof-of-concept trials and culminating in large-scale, randomized, controlled multicenter clinical studies, is fundamental to validating the effectiveness and safety of SIRT1-targeted strategies for human cardiac aging.

## 8. Conclusion

Cardiac aging is a multifaceted pathological process influenced by numerous factors, among which mitochondrial dysfunction plays a central role. This review systematically elaborates on the regulatory mechanisms of SIRT1 in maintaining mitochondrial quality control, encompassing processes such as mitochondrial biogenesis, energy metabolism, redox homeostasis, dynamics, and mitophagy. Additionally, the article summarizes potential SIRT1-targeted interventions and discusses the major challenges associated with translating these strategies into therapies for cardiac aging. In summary, targeting SIRT1 represents a promising therapeutic approach to modulate cardiac aging through the preservation of mitochondrial integrity.
